# Morphologic, genetic, and biogeographic continua among subspecies hinder the conservation of threatened taxa: the case of *Centaurea aspera* ssp. *scorpiurifolia* (Asteraceae)

**DOI:** 10.1038/s41598-022-04934-4

**Published:** 2022-01-18

**Authors:** Alfonso Garmendia, Hugo Merle, Marta Sanía, Carmelo López, María Ferriol

**Affiliations:** 1 Instituto Agroforestal Mediterráneo (IAM), Universitat Politècnica de València, Camino de Vera s/n, 46022 Valencia, Spain; 2 Departamento de Ecosistemas Agroforestales, Universitat Politècnica de València, Valencia, Spain; 3 Centro Para La Conservación Y Mejora de La Agrodiversidad Valenciana (COMAV), Universitat Politècnica de València, Valencia, Spain

**Keywords:** Genetics, Plant sciences

## Abstract

Subspecies are widely included as conservation units because of their potential to become new species. However, their practical recognition includes variable criteria, such as morphological, genetic, geographic and other differences. *Centaurea aspera* ssp. *scorpiurifolia* is a threatened taxon endemic to Andalusia (Spain), which coexists in most of its distribution area with similar taxa. Because of the difficulty to identify it using morphology alone, we aimed to sample all the populations cited as ssp. *scorpiurifolia* as exhaustively as possible, morphologically characterise them, and analyse their genetic structuring using microsatellites, to better understand difficulties when conserving subspecies. Three different *Centaurea* species were found which were easily identified. Within *C. aspera*, two genetic populations and some admixed individuals were observed, one including ssp. *scorpiurifolia* individuals and the other including individuals identified as subspecies *aspera*, *stenophylla*, and *scorpiurifolia*. A morphological continuum between these two genetic populations and a wide overlapping of their biogeographic distribution were also found. This continuum can affect the conservation of ssp. *scorpiurifolia* because of potential misidentifications and harmful effects of subspecific hybridization. Misidentifications could be partly overcome by using as many different traits as possible, and conservation priority should be given to populations representative of the ends of this continuum.

## Introduction

One of the most popular definition of species is that proposed by Mayr^1^ as “groups of actually or potentially interbreeding populations that are reproductively isolated from other such groups”. Since then, several concepts of species have been proposed although all of them share the correspondence of species with metapopulation lineages or gene pools^2^.

Species can display great genetic diversity that is often partitioned in local populations forming intraspecific units, even if these groupings can form hybrid zones at their geographical boundaries^3,4^. The potential that each intraspecific unit has to become reproductively isolated, cumulate ecologically relevant adaptations and finally become a new species justifies conservative efforts to protect them against extinction^5^. Furthermore, species with numerous intraspecific units that have alternative responses to environmental change may be less prone to extinction^6^. The contribution of the intraspecific biodiversity has led modern plant conservationists to consider intraspecific units as valuable conservation units. Policy frameworks include different names for these intraspecific units depending on legislation, although all of them recognize “subspecies” as a valid entity for protection and listing^4^. The International Union for the Conservation of Nature (IUCN), the Convention on International Trade in Endangered Species of Wild Flora and Fauna (CITES), and red lists of threatened species of many countries around the world nowadays include subspecies^7^.

However, criteria for practical subspecies recognition largely vary among taxonomists. They include the existence of a partial reproductive isolation, differentiated geographical distributions^8^, evolutionary divergence^9^, common and diagnosable phylogenetically acquired phenotypic characters, unique natural history^5^, genetic adaptation to local habitats^6^, and behavioural, physiological and phenological differences^3,6^. Thus, the practical implementation of conservation programs has been hindered by a lack of a uniform definition of subspecies. Criteria mentioned above commonly do not occur at the same time or in a regular order^2^. For example, in some cases molecular genetic characterization contradicts morphological descriptions, because neutral genetic markers and phenotypic traits are influenced by different evolutionary forces^10^. Delimitation of subspecies can also be especially problematic when hybridization among them occur, or when taxonomical adscriptions are not accurate or stable^4,5^. In such cases, genomic analysis combined with morphological and reproductive studies may be needed to provide a clear taxonomic frame.

*Centaurea aspera* L. (Asteraceae) is endemic to Western Mediterranean. It comprises five subspecies: ssp. *aspera*, ssp. *stenophylla* (Dufour) Nyman, ssp. *pseudosphaerocephala* (Shuttlew ex. Rouy) Gugler, ssp. *scorpiurifolia* (Dufour) Nyman, and ssp. *gentilii*^11,12^. Of these subspecies, ssp. *scorpiurifolia* is a threatened taxon, classified as “Endangered” according to IUCN criteria^13^. It is included in the red lists of Andalusia^14^ and Spain^15,16^.

Morphologically, *C. aspera* spp. *scorpiurifolia* (hereinafter ssp. *scorpiurifolia*) differs from *C. aspera* ssp. *aspera* (hereinafter ssp. *aspera*) in the leaf shape and size and in the stem branching, although the differentiation is frequently difficult in the field^11,17,18^.

Subspecies *scorpiurifolia* grows in shrublands or pine and cork oak forests on coastal sandy soils, between 0 and 1400 masl^14,15,18^. Some other morphologically similar *Centaurea* taxa, such as *C. aspera* ssp *stenophylla* (hereinafter ssp. *stenophylla*) and ssp. *aspera*, *C. pullata*, and *C. sphaerocephala*, have similar ecological requirements, and sometimes they may be found growing together [17, 18, pers. obs.: see Table 1].

**Table 1:** Origin of the sampled *Centaurea* populations in Andalusia. The field trip was based on the citations of the localities (a^25^; b^58^; c^59^; d^20^; e^60^; f^61^; g^62^; h^63^; i^26^; j^22^; k^23^, l^24^. Geographic position, number of sampled individuals for genetic analyses (Ng) and for morphologic analyses (Nm), and the tentative taxon to which the individuals belong are described.

Biogeographical province	Province	Locality/citation	Population	Observations	Ng	Nm	Tentative taxon
Gaditano-Onubo-Algarvish	Huelva	Between Lepe and Ayamonte/a	Not prospected				
	Cádiz	Around Vejer de la Frontera/a, b, c, d, e, f	Libreros, N36 17.541 W5 55.289	Abundant individuals that seemed *C. aspera* ssp. *aspera* but with wide leaves, on fixed coastal sand dunes with *Pinus pinea* and *Quercus suber*	3	3	*C. aspera* ssp. *aspera* (or *scorpiurifolia*)
			La Herradura, N36 22.983 W6 08.201		4	2	*C. aspera* ssp. *aspera* (or *scorpiurifolia*)
		Around Chiclana de la Frontera and Puerto Real/a, b, d, g, h	La Barrosa, N36 15.116 W5 56.427	*C. sphaerocephala* on fixed sand dunes, some with small capitula, and ssp. *aspera* with wide leaves	7	4	*C. sphaerocephala*
					4	2	*C. aspera* ssp. *aspera* (or *scorpiurifolia*)
		Around Algeciras/a, i	Pinar del Rey, N36 14.107 W5 23.937	In disturbed habitats on sandy soil with *P. pinea* and *Q. suber*	2	1	*C. sphaerocephala* *C. pullata* not sampled
Baetic		Around Grazalema/a	Not sampled	In a *P. pinea* forest, only *C. pullata*			*C. pullata*
	Málaga	Sierra Prieta/c	Not prospected				
Gaditano-Onubo-Algarvish		Marbella/personal observation	Marbella, N36 30.136 W4 48.268	Some *C. sphaerocephala* individuals on a road edge, on sandy soil	4	2	*C. sphaerocephala*
Baetic	Córdoba	Around Cabra/a	Not sampled	Olive tree orchards with *C. pullata*			*C. pullata*
	Granada	Around Sierras de Almijara, Tejeda and Alhama/a, c	Jayena, N36 56.968 W3 49.603	Ruderal vegetation and *Pinus halepensis* pine forest	2	2	*C. aspera* ssp. *aspera*
		Around Quéntar/a	Not prospected				
		Around Jete (Otívar, between Molvízar and Lobres), personal observation	Otívar, N36 48.483 W3 40.638	Along the road near fruit trees, in a slightly more humid habitat	9	3	*C. aspera* ssp. *scorpiurifolia*
			Molvízar, N36 46.914 W3 35.741	Along the road, close to the previous ssp. *scorpiurifolia* population	6	3	*C. aspera* ssp. *aspera*
		Around Sierra Nevada/j	Órgiva, N36 51.792 W3 28.309	Along the path edge in mountains with *Pinus halepensis* and almond trees	10	6	*C. aspera* ssp. *scorpiurifolia*
			Órgiva, N36 51.660 W3 29.205	Two *C. aspera* ssp. *aspera* individuals on the road edge, at a lower altitude	2	2	*C. aspera* ssp. *aspera*
Murciano-Almeriense	Almería	Around El Ejido/a,k,l	La Parra, N36 46.993 W3 03.835	Close to the village, some *C. aspera* ssp. *scorpiurifolia* along the road	13	5	*C. aspera* ssp. *scorpiurifolia*
		Tabernas desert—Sierra Alhamilla Natural Park/k	Alhamilla, N36 59.614 W2 24.618	Along the road, on the W and N slopes of mountains with *P. halepensis*	9	5	*C. aspera* ssp. *scorpiurifolia*
		Around Bédar (Cerro Tenderas)/l	Bédar, N37 11.965 W1 59.706	*C. aspera* ssp. *scorpiurifolia* individuals on the road edge	13	5	*C. aspera* ssp. *scorpiurifolia*
Baetic		Around Serón/k	Porteros, N37 20.908 W2 27.728	Along the road near Porteros and Serón, on a tip in Lúcar	5	3	*C. aspera* ssp. *stenophylla*
Castellano-Maestrazgo-Manchega		Sierra María—Los Vélez/k	Vélez-Rubio, N37 38.673 W2 03.984	Abundant along the roads, mountain paths and almond trees orchards	2	2	*C. aspera* ssp. *stenophylla*
	Murcia	Sierra de la Pila Regional Park/a	Not prospected				
		Cehegín/a	Not prospected				

Although it is a taxon relatively common in its ecological habitat, its distribution area is very narrow^15^. It is considered an endemic of Andalusia (Southern Spain), specifically of the Gaditano-Onubo-Algarvish biogeographical Province (Mediterranean Region)^17,19–21^. However, ssp. *scorpiurifolia* has also been cited in the Baetic and Murciano-Almeriense biogeographical Provinces, in Almería, Granada, and Málaga political provinces^18,22–24^.

Only recently, Invernón et al.^25^ also cited its presence in Huelva, Córdoba and Murcia, using mainly herbarium samples. Furthermore, Gil et al.^26^ and Devesa et al.^18^ consider that this taxon is also distributed in North Western Africa (Morocco). However, we have not found any detailed citation in Morocco, and phytogeographical studies comparing both regions only reported ssp. *scorpiurifolia* in the Iberian Peninsula^27^.

Given the difficulty of the morphological differentiation between the threatened ssp. *scorpiurifolia* and other related taxa which grow in sympatry, and the growing number of citations of its presence that may affect the implementation of the IUCN criteria^13^, we aimed: (i) to perform an exhaustive exploration of its distribution area and sample all possible representative *Centaurea* populations, (ii) to analyse the genetic structure of the populations found and the possible gene flow among them, and (iii) to morphologically characterize the genetically identified ssp. *scorpiurifolia* and related taxa observed in its cited distribution area. The results would be useful to manage this threatened subspecies and to throw light on the difficulties of conserving intraspecific taxa.

## Results

### Population sampling

**Figure 1:**
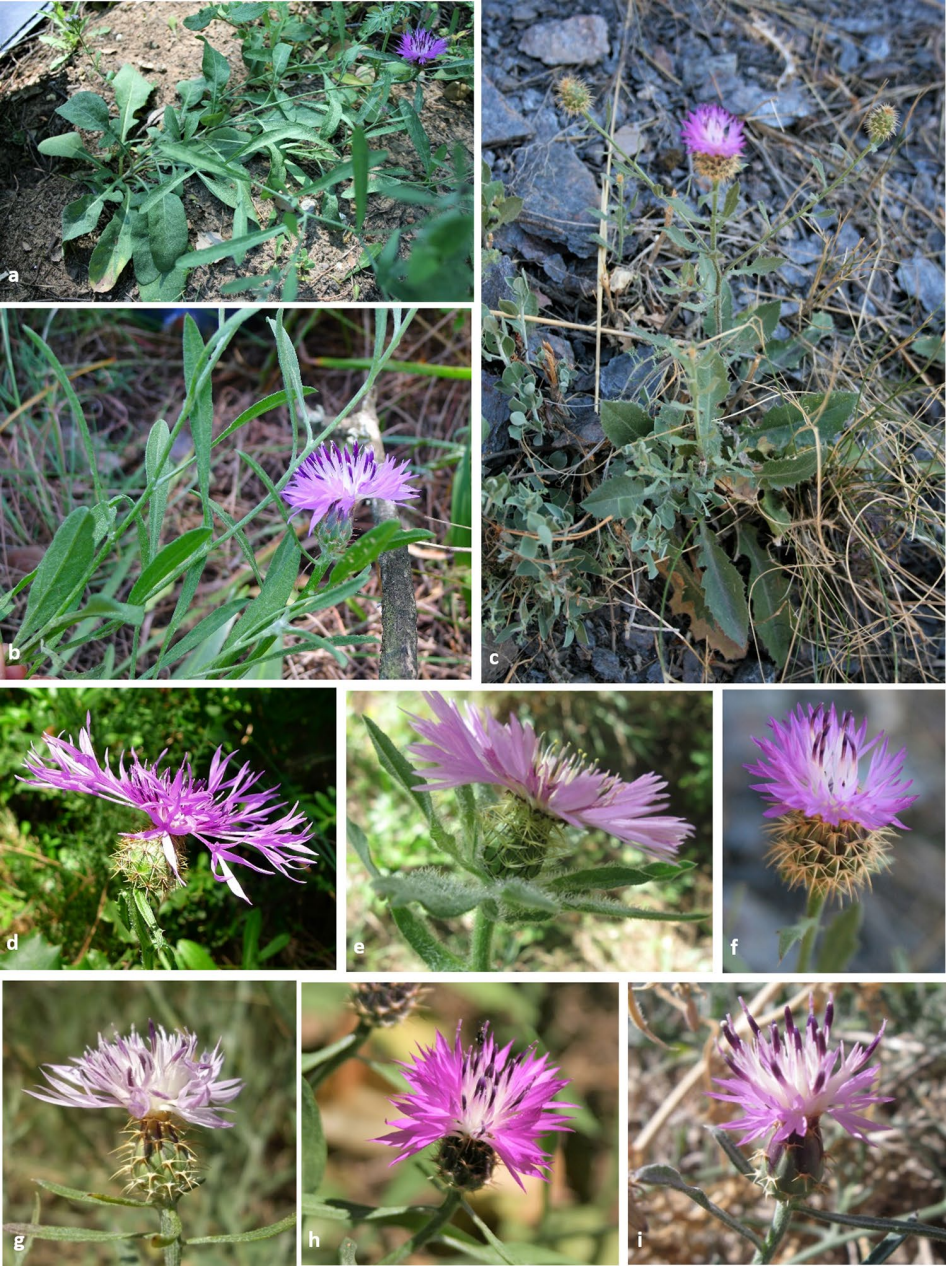
Plants of the observed *Centaurea* taxa in Andalusia and detail of their capitula. Plants: (**a**) Plant showing intermediate traits between *C. aspera* ssp. *aspera* and ssp. *scorpiurifolia* (Cádiz). (**b**) *C. aspera* ssp. *aspera* (Granada). (**c**) *C. aspera* ssp*. scorpiurifolia* (Almería). Capitula: (**d**) *C. sphaerocephala* (Cádiz). (**e**) *C. pullata* (Cádiz). (**f**) *C. aspera* ssp*. scorpiurifolia* (Almería). (**g**) *C. aspera* ssp. *aspera* (Granada). (**h**) Plant showing intermediate traits between *C. aspera* ssp. *aspera* and ssp. *scorpiurifolia* (Cádiz). (**i**) *C. aspera* ssp. *stenophylla* (Almería).

We were able to find most of the populations cited in the literature along the Andalusian coast and the mountain ranges near the sea from Cádiz, Málaga, Granada, and Almería political provinces (Table 1, see Supplementary Fig. S1 online). Subspecies *scorpiurifolia* was cited many times in Cádiz, in the Gaditano-Onubo-Algarvish biogeographical province. There, we found individuals whose morphological traits were intermediate between ssp. *aspera* and ssp. *scorpiurifolia*, as plants showed the typical traits of ssp. *aspera* but leaves were wider than 5 mms, which is characteristic of ssp. *scorpiurifolia* (Fig. 1). In the same biogeographical province, we also found *C. sphaerocephala* individuals forming pure populations (Málaga) or coexisting with the ssp. *aspera*/ssp. *scorpiurifolia* intermediate individuals (Cádiz – Pinar de la Barrosa) or with *C. pullata* (Cádiz, Pinar del Rey). In the most continental mountains of the Baetic biogeographical Province, around Grazalema (Cádiz) and Cabra (Córdoba), we only found *C. pullata* individuals. The most differentiated populations of ssp. *scorpiurifolia* were observed in Granada (Baetic Province) and Almería (Murciano-Almeriense Province), where the taxon has been cited consistently since 1980s. In Granada we found ssp. *aspera* and ssp. *scorpiurifolia* individuals growing in sympatry in Órgiva (Baetic Province). Interestingly, we found a new spp. *scorpiurifolia* population which was not cited previously in Otívar, 20 kms far from the nearest cited population in Órgiva. In the Baetic province, we also found some pure ssp. *aspera* populations (Jayena and Molvízar). We found three differentiated populations of ssp. *scorpiurifolia* in Almería. In two other locations which were far from the coast we found ssp. *stenophylla* individuals, in the Baetic and Castellano-Maestrazgo-Manchega Provinces.

From an ecological viewpoint, *C. sphaerocephala* only grows in the more humid and warmer areas of the studied area (Supplementary Table S2 online). *Centaurea aspera* ssp. *aspera* can develop in a wide range of habitats, from thermo-Mediterranean to meso-Mediterranean thermotypes, semiarid-dry to subhumid-humid ombrotypes, and from shrublands to oak forests vegetation domains. Subspecies *scorpiurifolia* has slightly warmer and drier requirements, growing in thermo-Mediterranean and from semiarid to dry-subhumid areas, in shrublands and holm oak forests vegetation domains. Subspecies *stenophylla* also has slightly drier requirements, but grows in colder habitats (upper thermo-Mediterranean to meso-Mediterranean thermotypes).

### Genetic characterization

Nine loci were scored for the 95 individuals of *Centaurea* belonging to the *Seridia* section sampled in Andalusia. Rates of genotyping errors were negligible. No significant linkage disequilibrium between loci was found in *C. sphaerocephala* (Ia = 0.16 with *P* = 0.15 and rbarD = 0.03 with *P* = 0.15) and *C. aspera* (Ia = 0.01 with *P* = 0.06 and rbarD = 0.002 with P = 0.06). Two microsatellite loci (CM17 and CA005) resulted in nonamplifying bands (null alleles) only in *Centaurea sphaerocephala*. These were identified because of the presence of large artefact bands that were not present in the control reactions. Median frequencies of null alleles in the remaining loci varied from 0.00 to 0.46, with a mean median frequency of 0.12, in *C. sphaerocephala*. Median frequencies of null alleles in the nine loci varied from 0.00 to 0.38, with a mean median frequency of 0.22, in *C. aspera*.

**Figure 2:**
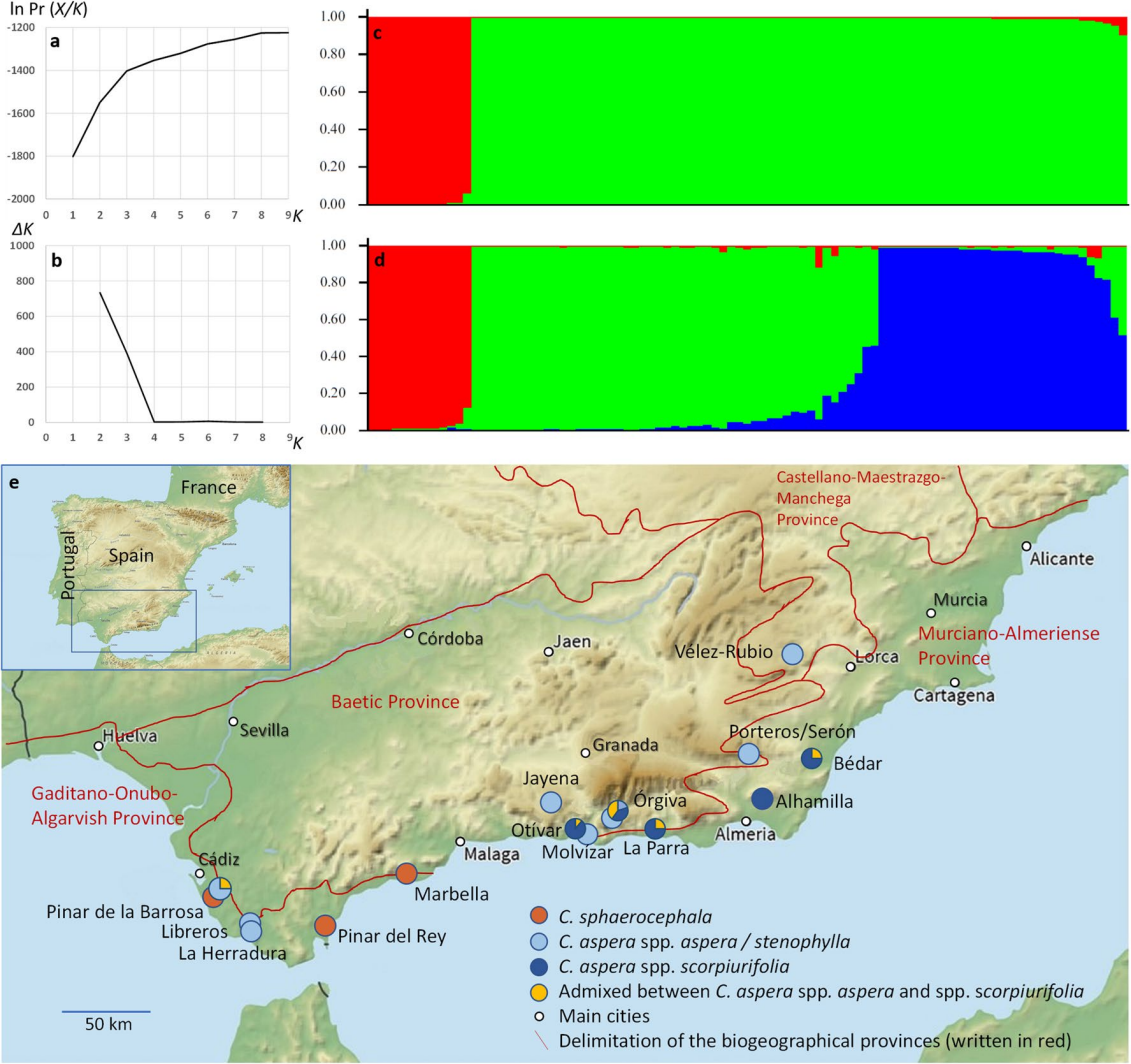
Clustering analysis for 95 *Centaurea* individuals of the *Seridia* section in Andalusia using STRUCTURE. (**a**) log-likelihood of the microsatellite data given *K* clusters obtained through 20 runs of the algorithm. (**b**) Evanno et al*.*’s *∆K* statistics. (**c**) Assignment tests for *K* = 2. Individual plants are represented by vertical bars. Red genetic cluster represents plants identified as *C. sphaerocephala*, while green cluster represents plants identified as *C. aspera*. (**d**) Assignment tests for *K* = 3. Red cluster represents plants identified as *C. sphaerocephala*, green cluster represents plants identified as *C. aspera* ssp. *scorpiurifolia*, and blue cluster those identified as *C. aspera* ssp. *aspera* or ssp. *stenophylla*. (**e**) Genetic constitution of the sampled locations based on STRUCTURE. Map was downloaded from https://mapswire.com/ (CC-BY 4.0), and modified using Microsoft Paint and Microsoft PowerPoint.

Diagrams of the STRUCTURE analysis representing log likelihood of the microsatellite data and Evanno et al.’s *∆K* statistics are shown in Fig. 2. Two peaks of *∆K* were found, with the maximized peak observed at *K* = 2, and a second peak at *K* = 3. This result suggested that the 95 sampled plants could be divided into two clusters, with no admixed individuals. Cluster 1 included 13 individuals which were morphologically identified as *C. sphaerocephala*, and cluster 2 included 82 individuals identified as *C. aspera*. The second peak found at *K* = 3 indicated that individuals could be further divided into three clusters (Fig. 2). Cluster 1 included the 13 *C. sphaerocephala* individuals previously detected. Cluster 2 included *C. aspera* individuals: 10 which were morphologically intermediate between ssp. *aspera* and ssp. *scorpiurifolia*, 10 identified as ssp. *aspera*, 7 identified as ssp. *stenophylla*, and two plants growing in Órgiva which were identified as ssp. *scorpiurifolia*. Hereinafter, cluster 2 will be called ssp. *aspera* cluster. Cluster 3 included 45 individuals identified as ssp. *scorpiurifolia*. Finally, with the arbitrary cutoff value of 80% ancestry for assignment, 8 *C. aspera* individuals were considered as admixed between clusters 2 and 3: one was identified as ssp. *aspera* from Chiclana de la Frontera, and 7 were identified as ssp. *scorpiurifolia* (two from Órgiva, one from Otívar, one from El Ejido and one from Bédar) (Fig. 2).

From a biogeographical viewpoint, all the individuals from Cádiz (Gaditano-Onubo-Algarvish Province) that were morphologically intermediate between ssp. *aspera* and ssp. *scorpiurifolia* belonged to ssp. *aspera* cluster. We only found genetically differentiated ssp. *scorpiurifolia* individuals in Granada and Almería political provinces, in the Baetic and Murciano-Almeriense Provinces respectively. However, most of the populations included some admixed individuals, which were more frequent in Órgiva (Baetic province), where individuals with clear morphological ssp. *aspera* traits and individuals with clear ssp. *scorpiurifolia* traits were coexisting.

**Figure 3:**
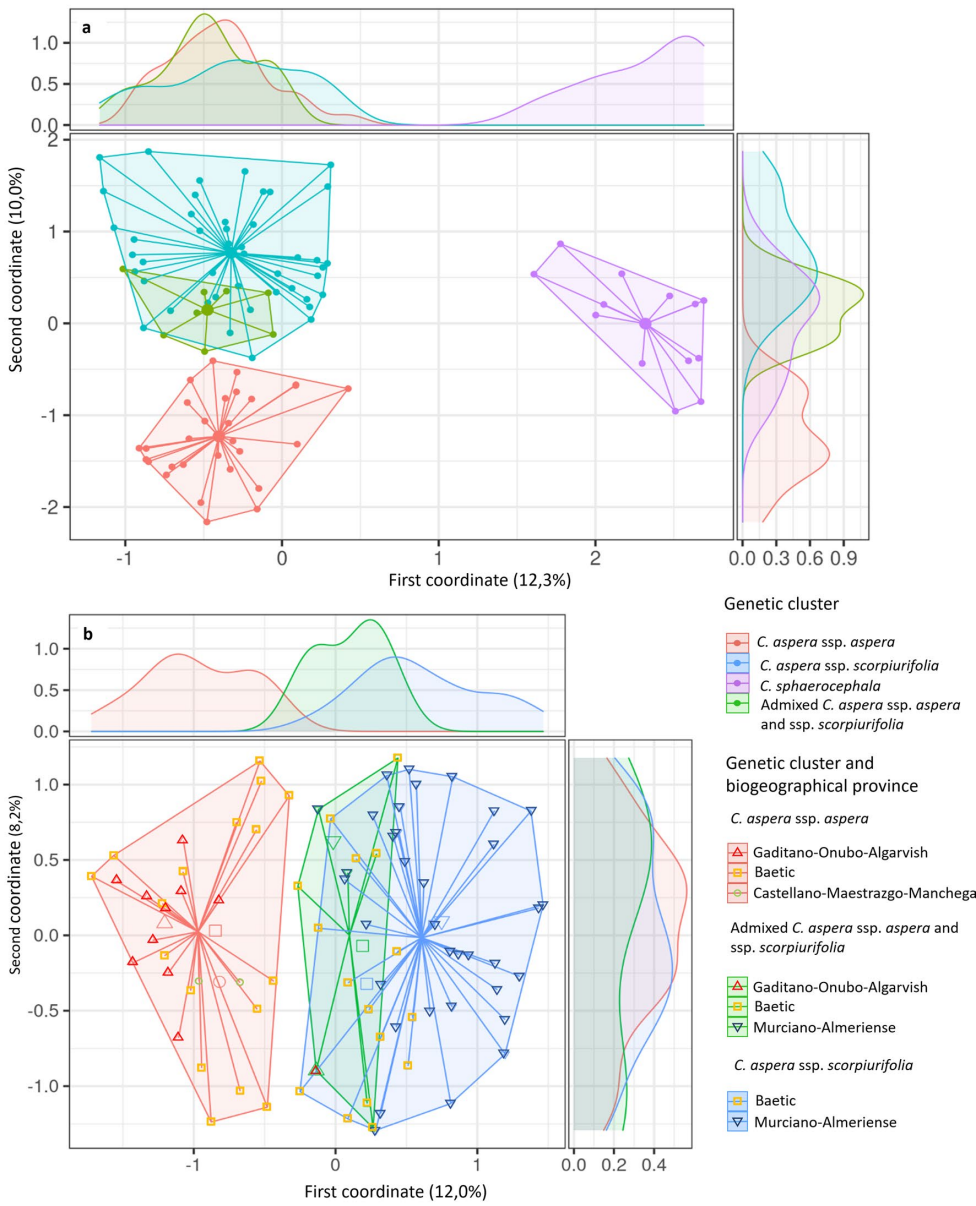
Principal Coordinates Analysis of *Centaurea* individuals of the *Seridia* section found in Andalusia, based on the genetic clusters detected using STRUCTURE. The percentage of the total variance accounted by each axis is shown in brackets. The densities of individuals within each cluster along the two axes are represented. (**a**) Ninety-five individuals (*C. sphaerocephala*, ssp. *aspera* cluster, *C. aspera* ssp. *scorpiurifolia,* and admixed individuals between the two latter). (**b**) Eighty-two individuals (ssp. *aspera* cluster, ssp. *scorpiurifolia,* and admixed individuals between them). Locations of the individuals according to biogeographical provinces are detailed.

The results of the PCoA using the nine microsatellite loci agreed with those obtained in STRUCTURE (Fig. 3a). The *C. sphaerocephala* individuals clustered separately from the *C. aspera* individuals. This grouping was also observed using only the seven microsatellite markers that resulted in amplifying bands in *C. sphaerocephala*, although some inferential and discriminatory power was lost (see Supplementary Fig. S3 online). Within *C. aspera*, individuals of the ssp. *aspera* cluster and those of ssp. *scorpiurifolia* were clearly separated, although the cluster of admixed individuals widely overlapped with that of ssp. *scorpiurifolia*, supporting a large introgression from ssp. *aspera* to ssp. *scorpiurifolia* (Fig. 3b). The PCoA also showed some grouping in the first coordinate according to biogeographical adscription of the ssp. *scorpiurifolia* individuals growing in the Baetic Province (Granada) and in the Murciano-Almeriense Province (Almería).

Genetic differentiation among taxa and populations were further analyzed through AMOVA (see Supplementary Table S4 online). 42.7% of genetic variation was found between species *C. sphaerocephala* and *C. aspera*. Accordingly, the value of differentiation *F*_*ST*_ between them was high (0.35). However, when we only considered clusters within *C. aspera*, only 19.2% of the variation was found between ssp. *aspera* cluster and ssp. *scorpiurifolia*, and *F*_*ST*_ was also lower but not negligible (0.13). In contrast to PCoA, no genetic differentiation was found among biogeographical provinces within taxon.

### Morphological characterization

*Centaurea sphaerocephala* differed from *C. aspera* in their significantly longer involucres, higher number of spines in each phyllary, and thicker upper leaves (Table 2). In addition, it showed hairy leaves in comparison with the glabrous leaves of *C. aspera*, although this trait was not completely uniform in all individuals (see Supplementary Table S5 online). Also, most *C. sphaerocephala* individuals displayed toothed upper leaves and all of them had lobed medium leaves which were mostly pinnate. However, the shape of upper and medium leaves was variable in *C. aspera* (see Supplementary Table S5 online).

**Table 2:** ANOVAs of the morphological variables among the *Centaurea* clusters obtained using STRUCTURE on genetic characterization (7 *C. sphaerocephala*, 20 *C. aspera* cluster, 19 *C. aspera* ssp. *scorpiurifolia*, and 4 admixed individuals between the two latter). Bonferroni correction, mean and standard error are indicated. Different letters indicate significant differences between clusters resulting from post-hoc Tukey HSD tests. Asterisks show variables used for PCA. For vegetative binary variables used for PCA, see Supplementary Table S5 online.

Variable	Acronym	p (ANOVA)	p (Bonf.Corr)	*C. sphaerocephala*		ssp. *aspera* cluster		*C. aspera* ssp. *scorpiurifolia*		Admixed *Centaurea*		
				Mean ± st. error	THDS	Mean ± st. error	THDS	Mean ± st. error	THDS	Mean ± st. error		THDS
Number of capitula in the main stem	NC	< 0.01	0.05	1.25 ± 0.17	-	8.42 ± 1.47	-	14.61 ± 1.88	-	18.50 ± 10.93		-
Capitulum total length (mm)	CL*	< 0.01	< 0.01	33.18 ± 1.74	a	25.52 ± 0.96	b	30.26 ± 0.38	a	28.56 ± 1.42		ab
Involucre total length (mm)	IL*	< 0.01	< 0.01	17.59 ± 0.68	a	12.73 ± 0.37	c	15.43 ± 0.33	b	13.29 ± 0.45		bc
Involucre maximum width (mm)	IW*	< 0.01	< 0.01	14.68 ± 1.60	a	9.05 ± 0.34	b	12.39 ± 0.37	a	11.44 ± 1.15		ab
Involucre roundness (IL/IW)	IR	0.03	0.34	1.25 ± 0.09	-	1.42 ± 0.04		1.26 ± 0.04		1.21 ± 0.17		
Spine maximum length (mm)	SL*	< 0.01	< 0.01	5.38 ± 0.53	a	2.23 ± 0.17	b	4.91 ± 0.24	a	2.68 ± 0.57		b
Number of spines per bract	NS*	< 0.01	< 0.01	11.71 ± 1.23	a	4.25 ± 0.33	c	6.47 ± 0.21	b	5.00 ± 0.82		bc
Number of inner flowers	NI	< 0.01	0.01	40.85 ± 9.03	-	21.75 ± 1.99	-	37.47 ± 2.65	-	35.75 ± 7.95		-
Number of outer flowers	NO	0.02	0.22	16.14 ± 1.87	-	13.28 ± 1.03	-	18.22 ± 1.19	-	14.38 ± 0.94		-
Plant height	PH	0.58	1.00	51.64 ± 2.28	-	54.65 ± 5.96	-	46.53 ± 2.19	-	56.00 ± 12.00		-
Plant width, larger diameter (cm)	PD	< 0.01	0.05	19.43 ± 3.91	-	56.15 ± 8.39	-	73.37 ± 8.80	-	133.50 ± 70.50		-
Plant width perpendicular diameter (cm)	PD2	< 0.01	0.10	12.43 ± 2.48	-	44.00 ± 6.74	-	64.21 ± 8.12	-	71.00 ± 30.00		-
Plant volume (HP x PD x PD2) (dm^3^)	VOL	0.17	1.00	15.63 ± 4.81	-	258.52 ± 101.13	-	316.44 ± 99.11	-	757.36 ± 643.71		-
Stem section (mm)	SS	0.02	0.47	3.23 ± 0.46	-	2.79 ± 0.27	-	4.05 ± 0.26	-	3.85 ± 0.69		-
Upper leaves: internode length (mm)	UI	0.46	1.00	9.09 ± 2.42	-	11.91 ± 2.20	-	14.70 ± 1.63	-	14.03 ± 4.51		-
Medium leaves: internode length (mm)	MI	0.71	1.00	22.93 ± 3.33	-	25.12 ± 2.57	-	22.94 ± 1.34	-	19.89 ± 3.33		-
Upper leaves: length with stalk (mm)	UL	0.77	1.00	26.30 ± 3.91	-	26.37 ± 2.07	-	28.17 ± 1.61	-	30.76 ± 4.90		-
Medium leaves: length with stalk (mm)	ML	0.07	1.00	79.84 ± 9.07	-	67.94 ± 5.20	-	57.27 ± 3.27	-	58.56 ± 9.29		-
Upper leaves: blade width (mm)	UW*	< 0.01	< 0.01	7.34 ± 0.98	a	4.13 ± 0.29	b	7.14 ± 0.54	a	5.91 ± 0.84		ab
Medium leaves: blade width (mm)	MW	0.01	0.25	28.73 ± 2.95	-	18.03 ± 1.53	-	22.95 ± 1.68	-	19.05 ± 3.26		-
Medium leaves: roundness (ML/MW)	MP*	< 0.01	< 0.01	2.83 ± 0.25	b	4.03 ± 0.29	a	2.58 ± 0.10	b	3.14 ± 0.32		ab
Upper leaves: thickness (mm)	UT*	< 0.01	< 0.01	0.58 ± 0.07	a	0.36 ± 0.02	b	0.35 ± 0.02	b	0.30 ± 0.04		b
Medium leaves: thickness (mm)	MT	< 0.01	0.02	1.06 ± 0.12	-	0.69 ± 0.05	-	0.70 ± 0.04	-	0.51 ± 0.05		-
Upper leaves: apical lobe length (mm)	UAL	0.61	1.00	26.30 ± 3.91	-	25.30 ± 1.94	-	27.88 ± 1.66	-	30.76 ± 4.90		-
Medium leaves: apical lobe length (mm)	MAL	0.83	1.00	36.92 ± 6.40	-	39.81 ± 4.67	-	43.00 ± 2.12	-	38.50 ± 7.52		-
Medium leaves: apical lobe width (mm)	MAW	< 0.01	0.14	21.68 ± 1.33	-	14.98 ± 1.66	-	22.31 ± 1.58	-	14.89 ± 1.49		-
Upper leaves: number of lobes	UNL	0.18	1.00	1.00 ± 0.00	-	1.00 ± 0.00	-	1.21 ± 0.14	-	1.50 ± 0.50		-
Medium leaves: number of lobes	MNL	0.23	1.00	5.71 ± 0.94	-	4.60 ± 0.89	-	3.13 ± 0.44	-	4.63 ± 1.31		-

Within *C. aspera*, ssp. *aspera* could be differentiated from ssp. *scorpiurifolia* in their significantly shorter and narrower capitula and involucres, their less and shorter spines on each phyllary, narrower upper leaves, and more elongated medium leaves (Table 2). In addition, most ssp. *aspera* individuals displayed entire upper and medium leaves, while most ssp. *scorpiurifolia* individuals had toothed upper and medium leaves (see Supplementary Table S5 online).

**Figure 4:**
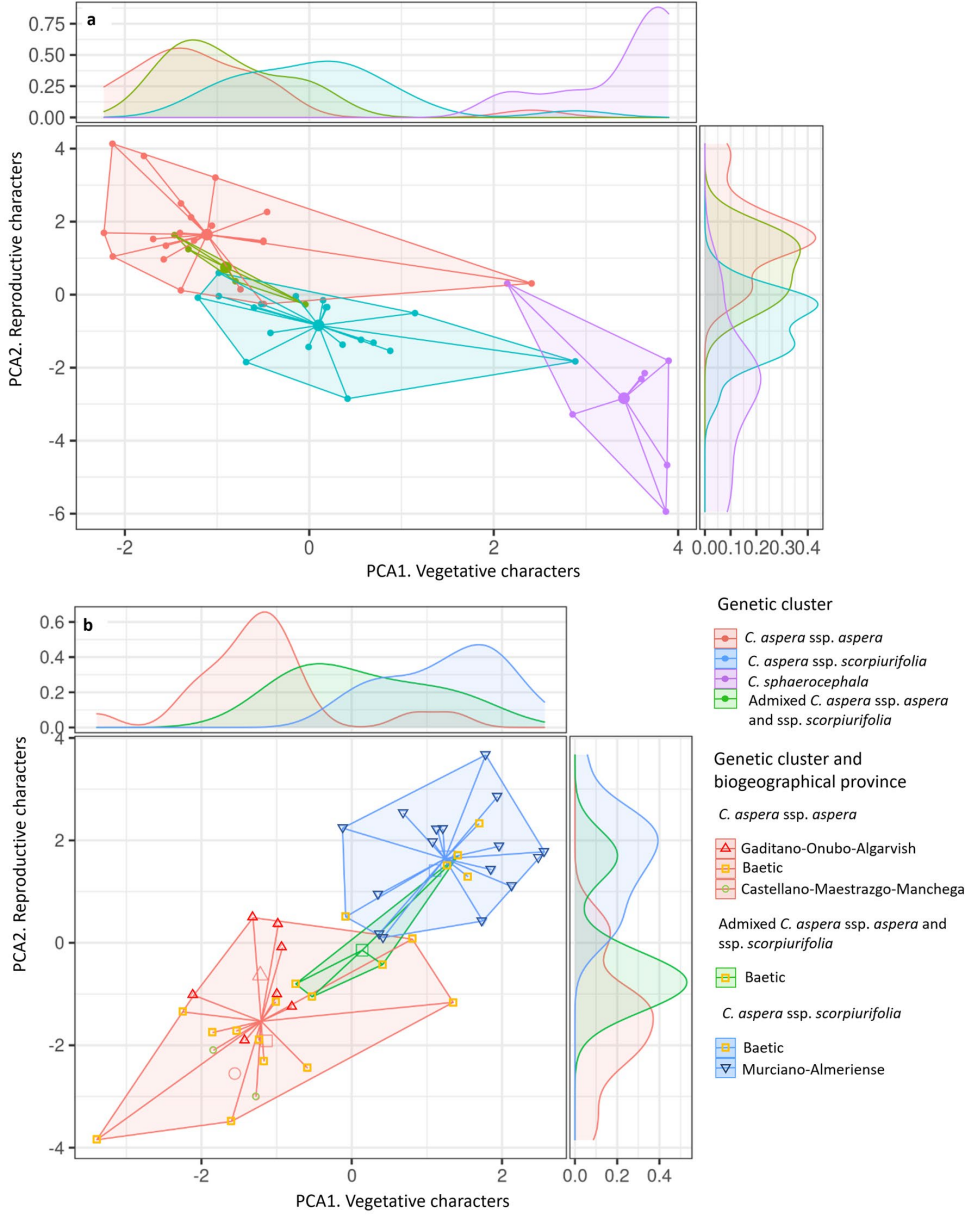
Two-dimensional plot based on the morphology of the sampled plants belonging to the clusters obtained using STRUCTURE on genetic characterization. The axes of the plot are the first principal components of the PCAs performed using vegetative and reproductive variables. The densities of individuals within each cluster along the two axes are represented. (**a**) Fifty individuals of *C. sphaerocephala*, ssp. *aspera* cluster, *C. aspera* ssp. *scorpiurifolia*, and 4 admixed between the two latter. (**b**) Forty-three *Centaurea aspera* individuals (ssp. *aspera* cluster, ssp. *scorpiurifolia*, and 4 admixed individuals between them).

The PCA performed on 50 *Centaurea* individuals using reproductive variables is shown in Supplementary Fig. S6 online. The first principal component accounted for 78.38% of the total variation, and clearly separated *C. sphaerocephala* from *C. aspera*. An even clearer separation of the two species was obtained in the PCA using vegetative variables (see Supplementary Fig. S7 online), in which the first principal component accounted for 43.30% of the total variation. Using both reproductive and vegetative characters, *C. sphaerocephala* appeared separated from *C. aspera*, although three individuals (one *C. sphaerocephala*, one ssp. *aspera* and one spp. *scorpiurifolia*) appeared in an intermediate position (Fig. 4a). Subspecies *scorpiurifolia* was more similar to *C. sphaerocephala* than to ssp. *aspera*. Within *C. aspera*, individuals formed a morphological continuum according to both vegetative and reproductive characters (Fig. 4b). Along this continuum, individuals of ssp. *aspera* cluster were grouped in one end and those of ssp. *scorpiurifolia* in the other end. Interestingly, admixed genetic individuals appeared also morphologically intermediate. Individuals from different biogeographical provinces within groups were morphologically similar.

### Correlation between morphological and genetic data

The distance matrices obtained using morphological and genetic data were significantly correlated (p value = 0.001). A high positive coefficient of correlation between them was found (r = 0.341).

## Discussion

Our results support the consideration of *Centaurea sphaerocephala* and *C. aspera* as distinct species, and ssp. *scorpiurifolia* as a distinct subspecies within the latter, using both morphological characters and molecular markers.

Regarding *C. sphaerocephala* and *C. aspera*, which belong to the same section *Seridia*, none of the sampled individuals was misidentified in the field according to both morphologic and genetic characters. Microsatellites were able to clearly separate both species and no admixed individuals were found, suggesting that gene flow is very limited between them. Morphological characters were also able to differentiate *C. sphaerocephala* and *C. aspera*, although one and two individuals respectively appeared to be morphologically very similar. However, not all the traits that discriminate both species were recorded, such as the presence/absence of pappus^18,28^, the arrangement of outer florets^11^, or the arrangement of the spines in one or more rows on the appendages of the phyllaries^17^. The consideration of these additional traits would probably morphologically discriminate both species without such overlapping. We only found *C. sphaerocephala* in the Gaditano-Onubo-Algarvish biogeographical Province, sometimes growing in sympatry with *C. aspera* without forming hybrids, while *C. aspera* had a wider distribution. Despite this clear taxonomic differentiation, in some locations where ssp. *scorpiurifolia* was cited, we only found *C. sphaerocephala*. This confusion also applied to *Centaurea pullata*. This is a morphologically very distinct species according mainly to phyllaries which belongs to a different section (sect. *Melanoloma*)^18^. In the locations that were furthest from the sea in which ssp. *scorpiurifolia* was cited only once^25^, we only found *C. pullata* individuals, suggesting a wider distribution area than ssp. *scorpiurifolia*, which is more restricted to areas near the sea. However, both *C. pullata* and *C. aspera* are included in the same genetic *Centaurea* Western Mediterranean clade^29^ and can cross forming morphologically intermediate sterile hybrids which are common in South Iberian Peninsula^30^, although we did not observe them. Consequently, this genetic similarity and the presence of intermediate forms can potentially lead to misidentifications.

Regarding the subspecies of *C. aspera*, our results showed that ssp. *scorpiurifolia* was genetically segregated from the cluster formed by the ssp. *aspera* cluster. This genetic differentiation was in accordance with the morphological analysis as assessed by the Mantel test, which is suggestive of an actual intraspecific structuring^31^.

However, although we observed individuals clearly adscribed to these different subspecies using genetic and morphological traits, we also found some intermediate individuals causing a continuum in both traits. In relation to the genetic traits, it has been shown that only few molecular loci are enough to delimit few lineages and estimate the proportion of admixture among populations^32,33^. Here, three individuals that were identified as a given subspecies appeared to be representatives of the other subspecies. Furthermore, we found a relatively high proportion of admixed individuals (9.75%) with a cutoff of 80%, which was previously used in other studies using microsatellites [i.e. 34]. However, this is an arbitrary cutoff which varies in different studies from 60% [i.e. 35] to 90% [i.e. 36]. Consequently, estimating admixture is a somewhat subjective task, and percentage of admixture may differ depending on thresholds. In relation with morphology, we also found some overlapping of individuals, even between those that were genetically considered as belonging to different subspecies but resulted to be morphologically very similar. In general, subspecies are recognized based on morphological observations alone, as was the case of ssp. *scorpiurifolia* until now. Usually, few distinct traits that show no overlap with other subspecies are used. Our results showed that this can be tricky, because of the presence of a morphological continuum. A highly variable morphology in well genetically defined subspecies was also found in other Asteraceae, such as *Arnica montana*^33^. In fact, Wiens^37^ argued that very large sample sizes may be required to be reasonably certain that a given trait is diagnostic at the desired level. The same occurred with the biogeographical distribution, which has also been suggested to delimit taxa boundaries^32^. In the Gaditano-Onubo-Algarvish province we could only find ssp. *aspera* and one admixed individual, while in the Murciano-Almeriense province, which is characterized by drier ombroclimates^19^, we could only find ssp. *scorpiurifolia* and some admixed individuals. However, both subspecies clearly coexisted in the Baetic province.

Even when using morphological and genetic analyses, which is unanimously suggested to infer taxa boundaries in combination with other traits^10,38^, the continuum among subspecies may persist^39^ as in our case. This overlapping in the different traits used may lead to different important issues in conservation. First, due to the presence of a continuum, some mistakes can be inevitable when determining the *C. aspera* subspecies, as has been shown in other plant groups^40^. Although genetic analyses can enhance their morphological identification, they do not provide a complete solution because of the presence of arbitrary thresholds when determining the number and nature of clusters^38^. Consequently, integration of morphology, genetics and biogeography should be applied to identify as much accurate as possible the ssp. *scorpiurifolia* populations. Second, taxonomic errors may inflate species distribution^41^. Here, we did not find ssp. *scorpiurifolia* in many cited locations especially in more continental areas, but other morphologically similar taxa. The fact that we could not locate the cited population is possible, but if this subspecies does not exist in these locations, its geographic range would be much narrower than expected. This issue is particularly sensitive in endangered taxa like ssp. *scorpiurifolia*, which has been classified as “endangered”^13^ because of its narrow areas of occupancy and occurrence, small population sizes, and continuing decline in the area and number of mature individuals^14,16^. Third, an overlapping also exists in the distribution areas of ssp. *aspera* and ssp. *scorpiurifolia* and this can lead to the presence of admixed populations. This is also the case with other plant subspecies that grow in sympatry^3^ and an increasing number of studies indicates that speciation can occur with gene flow and without geographical isolation in populations provided that these populations still display distinct morphology and genetic divergence^42^. Whether gene flow should be prevented to avoid genetic pollution of the endangered taxa or facilitated to increase its genetic diversity has long been debated in conservation biology^43^. At the intraspecific level, a beneficial effect of hybridization may be an increased genetic variation while avoiding types that might cause outbreeding depression as subspecies generally share alleles, whereas a harmful effect may be the loss of local adaptations^44^. In our case, we found populations with different degrees of admixture, many pure populations of ssp. *aspera*, and only one pure population of ssp. *scorpiurifolia*. This may indicate the presence of hybrid swarms that can extend to all populations in a relatively short time lapse. The geographically restricted remaining non introgressed ssp. *scorpiurifolia* population from Alhamilla should therefore be preserved from hybridization with the highest priority, although admixed populations should also be protected in the hope that they will fill the ecological role of the threatened subspecies^44^.

Our results support that even between well differentiated subspecies, like *Centaurea* ssp. *aspera* and ssp. *scorpiurifolia*, a continuum in morphology, genetic composition, and biogeographical distribution may be present. This can lead to difficulties in conserving threatened subspecies, which may be related with misidentifications and hybridization. Misidentifications could be partly overcome by using as many different traits as possible, and conservation priority should be given to populations that are representative of the ends of this continuum.

## Methods

### Population sampling and plant material

Prior to the field sampling trips, a detailed list of citations of ssp. *scorpiurifolia* was performed based on published literature (Table 1) and websites such as GBIF (Global Biodiversity Information Facility: https://www.gbif.org), Anthos (Spanish Plants Information System: http://www.anthos.es), SIVIM (Iberian and Macaronesian Vegetation Information System: http://www.sivim.info), and REDIAM (Environmental information Net of Andalusia: http://www.juntadeandalucia.es). In spring 2015, we performed an exhaustive sampling of the cited populations, considering especially the geographical coordinates and details of their locations, and we recorded the presence of spp. *scorpiurifolia* and related taxa (ssp. *aspera*, ssp. *stenophylla*, *C. sphaerocephala*, belonging to sect. *Seridia*; and *C. pullata*, belonging to sect. *Melanoloma*) that are morphologically similar. We visited the coast and the mountain ranges near the sea, in the Andalusian political provinces of Cádiz, Málaga, Granada, Córdoba, and Almería (Table 1). We did not visit Murcia and Huelva, where the taxon has only been cited once. We further extended the field expeditions to any potential habitats of ssp. *scorpiurifolia*. We tentatively identified the *Centaurea* taxa found in the cited localities using local and other floras and based on our own field experience. Geographical coordinates of all sampled populations were recorded using G.P.S. (Garmin eTrex Vista HCx).

Young leaf samples of 95 individuals separated by at least 5 m were collected for further genetic analysis (Table 1). Eleven individuals from Cádiz that were identified as ssp. *aspera* because of their entire upper leaves, although they were more than 5 mms wide (which is characteristic of ssp. *scorpiurifolia*), 10 ssp. *aspera* from Granada, 54 ssp. *scorpiurifolia* from Granada and Almería, 7 ssp. *stenophylla* from Almería, and 13 *C. sphaerocephala* from Cádiz and Málaga were included. Leaves were transported in a cooler and frozen plant tissues were stored at -80ºC.

Furthermore, 50 of these sampled individuals were morphologically characterized in the field to not damage the plant, including 7 that displayed traits of both ssp. *aspera* and ssp. *scorpiurifolia*, 7 ssp. *aspera*, 24 ssp. *scorpiurifolia*, 5 spp. *stenophylla*, and 7 *C. sphaerocephala* (Table 1). Voucher specimens of single branches growing on well-developed ssp. *scorpiurifolia* plants were also collected, dried by being pressed in absorbent paper, stored at room temperature, and kept in the Herbarium of the Universitat Politècnica de València (VALA): VALA 9581 (from Bédar), VALA 9582 (Alhamilla), VALA 9583 (La Parra), and VALA 9584 (Órgiva). Voucher specimens were identified by Alfonso Garmendia, Hugo Merle and María Ferriol.

All the *C. pullata* individuals found were not sampled because of its clear taxonomic adscription based on capitula and leaf traits.

### Genetic characterization using microsatellites

Genomic DNA isolation and amplification of nine microsatellite loci specifically developed for *Centaurea* were performed following Ferriol et al.^45^. Separation of the amplified fragments was carried out using a QIAxcel DNA High Resolution Kit (1200) (QIAGEN), which provides up to a 2 bp resolution when used with the OM700 method on fragments that range 100–500 bp in size, and analysis was performed using the BioCalculator software for the QIAxcel system, following Dean et al.^46^. To confirm the reproducibility of microsatellite fragments, PCR reactions were replicated in approximately 70% of the individuals for each locus, which were selected to maximize the genotypic diversity. Reamplified fragments were separated on polyacrylamide gels following Ferriol et al.^45^.

### Morphological characterization

The morphological characterization of 50 flowering complete individuals (with caulinar leaves, stems, and capitula but without basal leaves that were already dry) was accomplished in the field during spring to reveal differences between taxa, or any other morphological variation patterns. Table 2 shows the list of the characters that include those traditionally used for differentiation of the *Centaurea aspera* subspecies as can be found in determination keys and floras, as well as several that are potentially useful for distinction of taxa of the sect. *Seridia*. A total of 28 quantitative variables were evaluated: 9 corresponded to reproductive traits and 19 to vegetative traits. Seven qualitative characters were also evaluated (see Supplementary Table S5 online).

### Statistical analysis

#### Microsatellite analysis

Linkage disequilibrium between microsatellite loci in *C. sphaerocephala* and *C. aspera* was investigated by testing significance of the index of association, Ia, and of its standardized alternative rbarD^47^, with 999 randomizations using the R package Poppr^48^. Frequency of null alleles in *C. sphaerocephala* and *C. aspera* was estimated in R using package PopGenReport^49^.

The population structure of the sampled *Centaurea* individuals was estimated using software STRUCTURE v2.3.4^50^, with the admixture model and the correlated allele frequencies between populations options. To estimate the number of populations (*K*), we ran STRUCTURE with varying *K* values, ranging from 1 to 9. Each run consisted of one million burnin iterations and 500 000 data collection iterations. Each value of *K* was evaluated using 20 independent Markov chain Monte Carlo replicates. The number of clusters was inferred following Evanno et al.^51^, based on the values of *ΔK* for each value of *K* (except for *K* = 1 and the maximum *K* tested). We attributed a plant to a given cluster when the proportion of its genome in the cluster (*q*_*K*_) was higher than an arbitrary cutoff value of 0.8. Otherwise, the plant was classified as admixed.

A Principal Coordinates Analysis (PCoA) was performed in order to analyse relationships among the three observed genetic clusters using the adegenet package^52^ in R. A hierarchical partition of the genetic variation among species and populations from different biogeographical provinces was determined with analyses of molecular variance (AMOVAs) with 1000 permutations using the pegas package^53^. As AMOVA is not able to detect admixed populations in contrast to STRUCTURE, those admixed individuals found in STRUCTURE between genetic clusters were assigned to a given cluster according to its highest *q*_*K*_. In addition, genetic differentiation among populations for the highest levels of the hierarchy was also estimated following the suggestions of Whitlock^54^ for within species purposes when using microsatellite markers, i.e. F_ST_, and H_S_ (the heterozygosity within populations) that can be used for other calculations.

#### Morphological analysis

Analyses were carried out in R using the packages mass^55^ and agricolae^56^. As a first step, reproductive and vegetative characters of the genetic clusters were analysed separately. Qualitative vegetative traits were transformed into binary characters. Descriptive statistical measures (mean, standard deviation and error, etc.) were computed for the quantitative variables. ANOVAs and posthoc Tukey HSD comparisons among genetic clusters were calculated for all the quantitative variables. Bonferroni correction was applied to the ANOVAs significance to correct the effect of several repeated analyses.

A Principal Component Analysis (PCA) was performed using the variables that showed significant differences in the previous ANOVAs (*p* < 0.01) (Table 2, Supplementary Table S5 online). The frequencies of the binary variables UDB, MBM and MLM showed no significant differences among clusters, and were not included in the analysis. Two PCAs were firstly performed using the vegetative and the reproductive characters separately. Subsequently, the first principal component of each PCA was used as axis in a two-dimensional plot. The density of individuals of each genetic cluster was represented along each axis.

#### Correlation between morphological and genetic distances

A Mantel test was performed using package ade4^57^ in R to estimate the correlation between the pairwise Euclidean distances obtained from morphologic and genetic data considering the 50 individuals that were morphologically characterized and running 999 permutations.

### Permissions for collecting plant specimens

Although included in the IUCN Red Lists from Spain and Andalusia as stated in the Introduction, *C. aspera* ssp. *scorpiurifolia* is not under protection in any legislation. All the samples have been collected in non-protected areas, excepting the location of Alhamilla, included in the “Paraje Natural Sierra de Alhamilla”. However, the Natural Resources Ordinance Plan (Plan de Ordenación de los Recursos Naturales, PORN) (https://www.juntadeandalucia.es/medioambiente/portal_web/web/temas_ambientales/espacios_protegidos/planificacion/porn/2016_parajes_al_hu_ja/4_anexo9_porn_alhamilla_tabernas_boja.pdf), was addressed on 23rd December 2016, more than one year after our field trip, so no permission was needed. All the samples were collected following the IUCN Policy Statement on Research Involving Species at Risk of Extinction. We used non-lethal sampling methods (small samples of leaves for genetic characterization), we morphologically characterize plants in the field, and we took a single branch of a well-developed plant for herbarium specimens.

## Supplementary Information

Supplementary Information.

## Data Availability

The datasets generated during and/or analysed during the current study are available from the corresponding author on reasonable request.
