# Supplementary material for: Morphologic, genetic, and biogeographic continua among subspecies hinder the conservation of threatened taxa: the case of Centaurea aspera ssp. scorpiurifolia (Asteraceae)
Source: Sci Rep. 2022 Jan 18;12:932. doi: 10.1038/s41598-022-04934-4 (PMC8766572; doi:10.1038/s41598-022-04934-4)

Morphologic, genetic, and biogeographic continua among subspecies hinder the conservation of threatened taxa: the case of *Centaurea aspera* ssp. *scorpiurifolia* (Asteraceae)

Alfonso Garmendia[1**], Hugo Merle[2**], Marta Sanía[2], Carmelo López[3], María Ferriol[1*]

Supplementary Figure S1. Location of the sampled *Centaurea* populations based on the citations of *C. aspera* ssp. *scorpiurifolia* in the literature. The taxonomical adscription was based on floras of Spain and Andalusia and on our own experience. Map was downloaded from https://mapswire.com/ (CC-BY 4.0), and modified using Microsoft Paint and Microsoft PowerPoint.

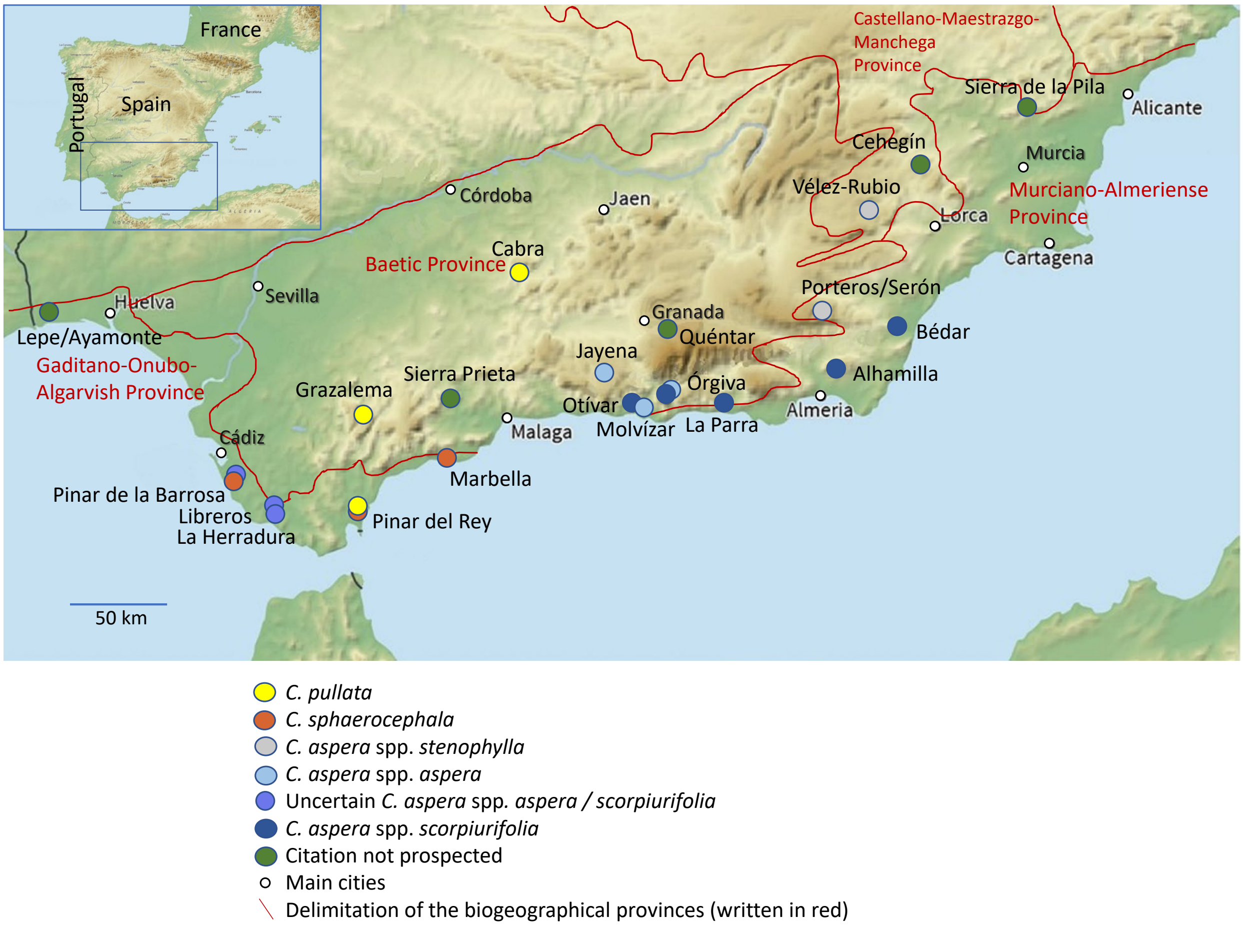

Supplementary Table S2. Ecology (bioclimate, substrate, vegetation, biogeography and localities) for *Centaurea sphaerocephala*, *C. aspera* ssp. *aspera*, ssp. *stenophylla*, and ssp. *scorpiurifolia*. Data were collected from REDIAM (Environmental information Net of Andalusia: http://www.juntadeandalucia.es)

Vegetation series	Vegetation domain	Thermotype	Ombrotype	Litology / soils	Biogeography	Localities	Taxon
Tamo communis-Oleeto sylvestris S.	Community with wild olive trees	Thermo mediterranean	Subhumid-humid	Vertic soils	Baetic, Gaditano-Onubo-Algarvish	Libreros, La Barrosa, Pinar del Rey (Cádiz)	*C. sphaerocephala* *C. aspera* spp. *aspera* (or *scorpiurifolia*)
Oleo-Querceto suberis S.	Cork oak forests	Thermo mediterranean	Subhumid-humid	Sabulicolous soils from sandstones	Gaditano-Onubo-Algarvish	La Herradura (Cádiz), Marbella (Málaga)	*C. sphaerocephala* *C. aspera* spp. *aspera* (or *scorpiurifolia*)
Smilaco mauritanicae-Quercetum rotundifoliae S.	Holm oak forests	Thermo mediterranean	Dry-subhumid	Basophilous	Baetic, Murciano-Almeriense	Órgiva (Granada); La Parra (Almería)	*C. aspera* ssp. *scorpiurifolia* and ssp. *aspera*
Bupleuro gibraltarici-Pistacieto lentisci S.	Shrubland with *Pistacia lentiscus*	Thermo mediterranean	Semiarid-dry	Indifferent soil but prefers basophilous.	Baetic, Murciano-Almeriense	Otívar, Molvízar (Granada); Alhamilla (Almería)	*C. aspera* ssp. *scorpiurifolia* and ssp. *aspera*
Chamaeropo humilis-Rhamneto lycioidis S.	Shrubland with *Pistacia lentiscus*	Upper thermo mediterranean	Semiarid	Basophilous	Murciano-Almeriense, Baetic	Bédar, Porteros (Almería)	*C. aspera* ssp. *scorpiurifolia* and ssp. *stenophylla*
Paeonio coriaceae-Querceto rotundifoliae S.	Holm oak forests	Meso mediterranean	Dry-subhumid	Basophilous	Baetic, Castellano-Maestrazgo-Manchega	Jayena (Granada) and Vélez-Rubio (Almería)	*C. aspera* spp. *aspera* and ssp. *stenophylla*

Supplementary Figure S3. Principal Coordinates Analysis of 95 *Centaurea* individuals of the *Seridia* section found in Andalusia, based on the genetic clusters detected using STRUCTURE (*C. sphaerocephala*, ssp. *aspera* cluster, *C. aspera* ssp. *scorpiurifolia,* and admixed individuals between the two latter) and using only the 7 microsatellite loci that resulted in amplifying bands in both species. The percentage of the total variance accounted by each axis is shown in brackets. The densities of individuals within each cluster along the two axes are represented.

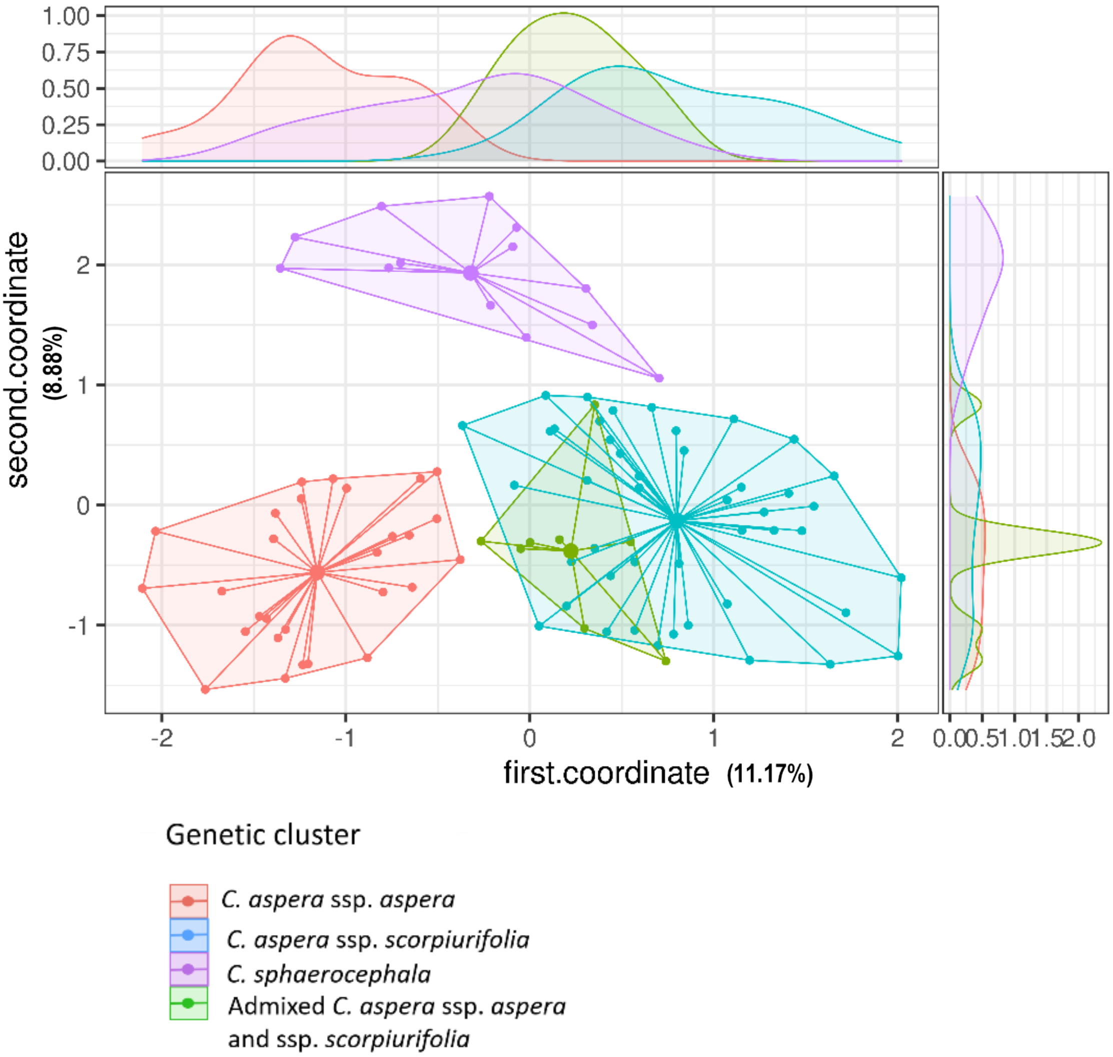

Supplementary Table S4. Analyses of molecular variance (AMOVAs) for taxa (*Centaurea sphaerocephala*, ssp *aspera* cluster and *C. aspera* ssp. *scorpiurifolia*) and for biogeographical provinces and sectors.

Grouping	Source of variation	d.f.	SSD	MSD	Variance component	Variance (%)	F_{ST}	H_S
Centaurea species (CSP, CA) using all 9 loci	Among species	1	56.89	56.89	2.39	42.7	0.35	0.49
	Within spp.	93	298.28	3.21	3.21	57.3		
Centaurea species (CSP, CA) using only the 7 microsatellite loci that resulted in amplifying bands in both species	Among species	1	29.75	29.75	1.19	28.1	0.23	0.63
	Within spp.	93	283.67	3.05	3.05	72.9		
STRUCTURE *Centaurea* populations (CSP, CAA, CAS) using all 9 loci	Among taxa	2	87.43	43.72	1.46	33.5	0.33	0.48
	Within taxa	92	267.74	2.91	2.91	76.5		
STRUCTURE *Centaurea* populations (CSP, CAA, CAS) using only the 7 microsatellite loci that resulted in amplifying bands in both species	Among taxa	2	60.20	30.10	0.98	26.3	0.25	0.60
	Within taxa	92	253.22	2.75	2.75	73.7		
STRUCTURE *Centaurea aspera* populations (CAA, CAS)	Among ssp.	1	30.51	30.51	0.71	19.2	0.13	0.50
	Within ssp.	80	238.36	2.98	2.98	81.8		
STRUCTURE *Centaurea aspera* populations (CAA, CAS) and biogeographical provinces	Among ssp.	1	30.51	30.51	0.70	19.2		
	Among provinces within ssp.	2	5.51	2.75	-0.02	0		
	Within provinces	78	232.85	2.99	2.99	81.8		

CA: *C. aspera*, CAA: ssp. *aspera* cluster (*C. aspera* ssp. *aspera/stenophylla*), CAS: *C. aspera* ssp. *scorpiurifolia,* CSP: *C. sphaerocephala*

Supplementary Table S5. Values of qualitative morphological vegetative variables in the individuals of the *Centaurea* clusters obtained using STRUCTURE on genetic characterization (7 *C. sphaerocephala*, 20 *C. aspera* ssp. *aspera/stenophylla*, 19 *C. aspera* ssp. *scorpiurifolia*, and 4 admixed individuals between the two latter).

	UDU* (hairiness of upper face of upper leaves)		
	glabrous	hairy	
C. sphaerocephala	1	6	
C. aspera ssp. *aspera/stenophylla*	17	3	
C. aspera ssp. *scorpiurifolia*	18	1	
Admixed	4	0	
	MDU* (hairiness of upper face of medium leaves)		
	glabrous	hairy	
C. sphaerocephala	1	6	
C. aspera ssp. *aspera/stenophylla*	19	1	
C. aspera ssp. *scorpiurifolia*	19	0	
Admixed	4	0	
	UDB (hairiness of back face of upper leaves)		
	glabrous	hairy	
C. sphaerocephala	0	7	
C. aspera ssp. *aspera/stenophylla*	16	4	
C. aspera ssp. *scorpiurifolia*	14	5	
Admixed	3	1	
	MDB* (hairiness of back face of medium leaves)		
	glabrous	hairy	
C. sphaerocephala	0	7	
C. aspera ssp. *aspera/stenophylla*	19	1	
C. aspera ssp. *scorpiurifolia*	15	4	
Admixed	4	0	
	UBM* (blade margin of upper leaves)		
	entire	toothed	lobed
C. sphaerocephala	1	6	0
C. aspera ssp. *aspera/stenophylla*	18	2	0
C. aspera ssp. *scorpiurifolia*	1	18	0
Admixed	2	2	0
	MBM (blade margin of medium leaves)		
	entire	toothed	lobed
C. sphaerocephala	0	0	7
C. aspera ssp. *aspera/stenophylla*	4	2	14
C. aspera ssp. *scorpiurifolia*	0	5	14
Admixed	0	1	3
	MLM (shape of lobes of medium lobed leaves)		
	lyrate	pinnate	
C. sphaerocephala	1	6	
C. aspera ssp. *aspera/stenophylla*	7	7	
C. aspera ssp. *scorpiurifolia*	12	2	
Admixed	0	3	

* Variables used for PCA

Supplementary Figure S6. Biplot of the two first principal components of the Principal Component Analysis (PCA) using reproductive variables, showing the individual positions of the sampled plants belonging to the clusters obtained using STRUCTURE on genetic characterization (*C. sphaerocephala*, ssp. *aspera* cluster, *C. aspera* ssp. *scorpiurifolia*, and admixed individuals between the two latter). The densities of individuals within each cluster along the two axes are represented.

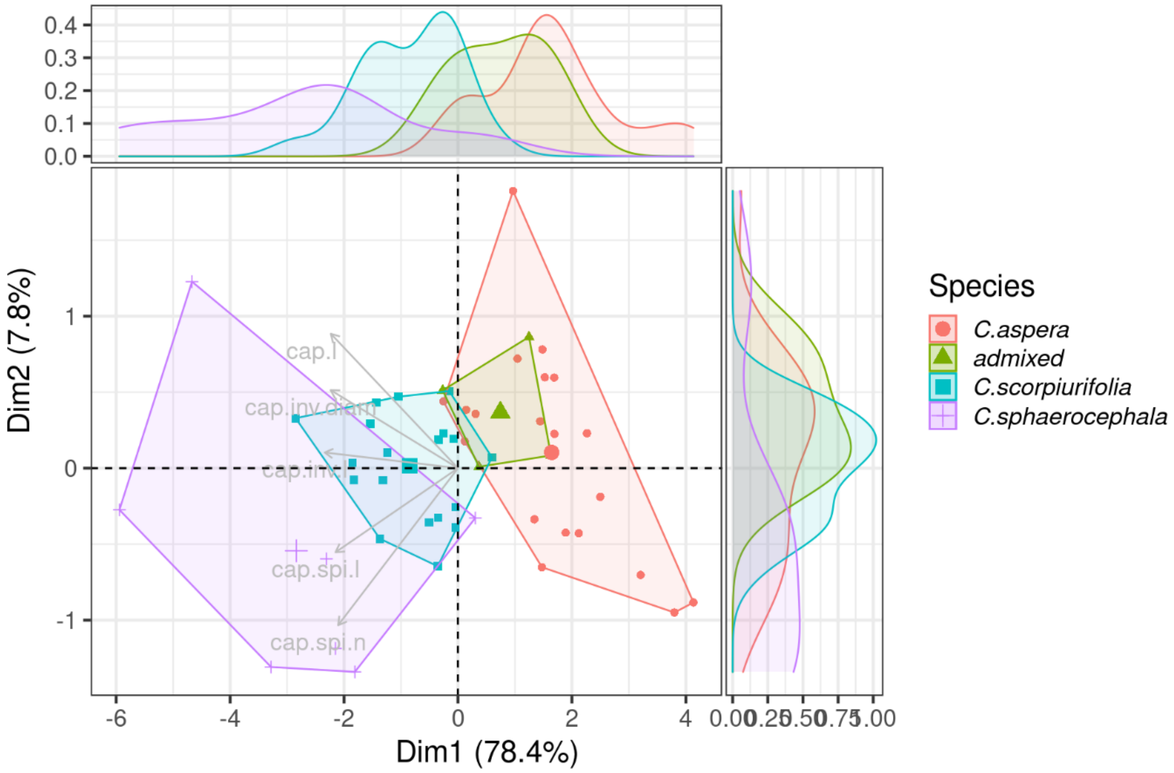

Supplementary Figure S7. Biplot of the two first principal components of the Principal Component Analysis (PCA) using vegetative variables, showing the individual positions of the sampled plants belonging to the clusters obtained using STRUCTURE on genetic characterization (*C. sphaerocephala*, ssp. *aspera* cluster, *C. aspera* ssp. *scorpiurifolia*, and admixed individuals between the two latter). The densities of individuals within each cluster along the two axes are represented.

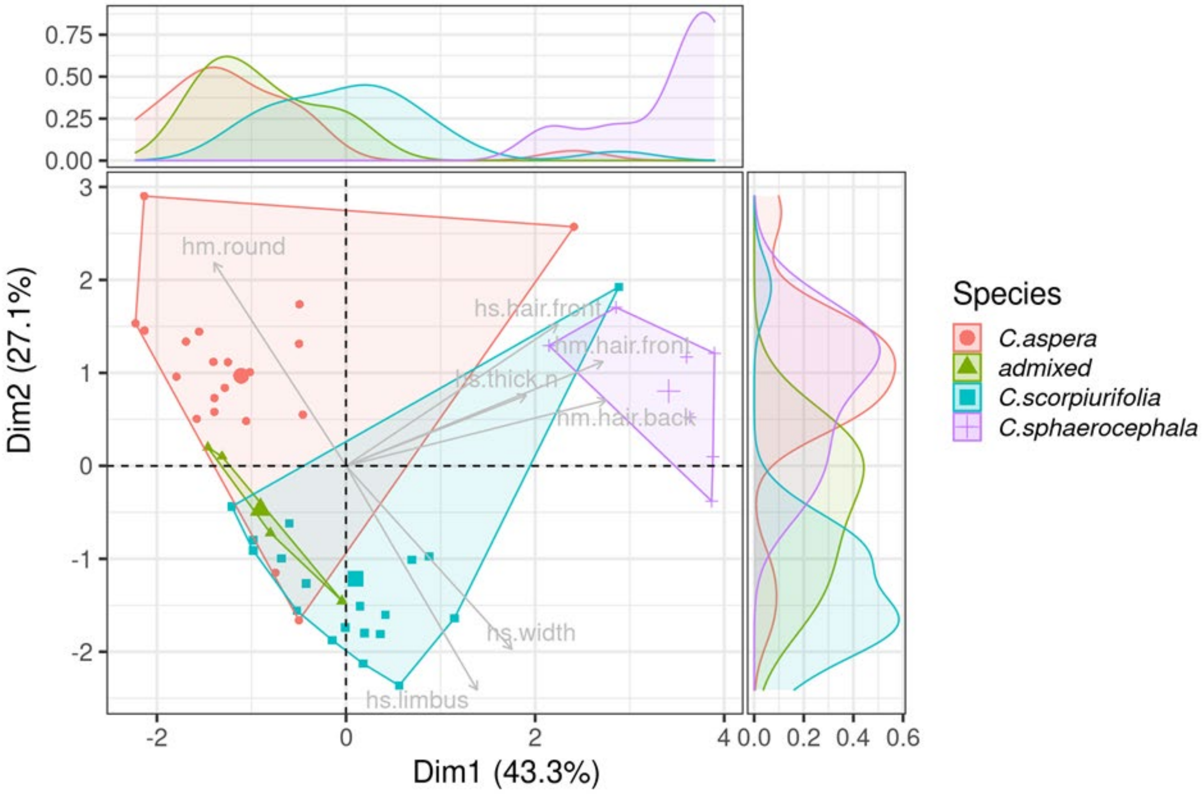

Supplement: Supplementary file 1 — Supplementary Information. [file 41598_2022_4934_MOESM1_ESM.pdf]
